# Bitter Taste Receptors Influence Glucose Homeostasis

**DOI:** 10.1371/journal.pone.0003974

**Published:** 2008-12-18

**Authors:** Cedrick D. Dotson, Lan Zhang, Hong Xu, Yu-Kyong Shin, Stephan Vigues, Sandra H. Ott, Amanda E. T. Elson, Hyun Jin Choi, Hillary Shaw, Josephine M. Egan, Braxton D. Mitchell, Xiaodong Li, Nanette I. Steinle, Steven D. Munger

**Affiliations:** 1 Department of Anatomy & Neurobiology, University of Maryland School of Medicine, Baltimore, Maryland, United States of America; 2 Senomyx, Inc., San Diego, California, United States of America; 3 National Institute on Aging/NIH, Baltimore, Maryland, United States of America; 4 Department of Medicine, Division of Endocrinology, University of Maryland School of Medicine, Baltimore, Maryland, United States of America; Duke Unviersity, United States of America

## Abstract

TAS1R- and TAS2R-type taste receptors are expressed in the gustatory system, where they detect sweet- and bitter-tasting stimuli, respectively. These receptors are also expressed in subsets of cells within the mammalian gastrointestinal tract, where they mediate nutrient assimilation and endocrine responses. For example, sweeteners stimulate taste receptors on the surface of gut enteroendocrine L cells to elicit an increase in intracellular Ca^2+^ and secretion of the incretin hormone glucagon-like peptide-1 (GLP-1), an important modulator of insulin biosynthesis and secretion. Because of the importance of taste receptors in the regulation of food intake and the alimentary responses to chemostimuli, we hypothesized that differences in taste receptor efficacy may impact glucose homeostasis. To address this issue, we initiated a candidate gene study within the Amish Family Diabetes Study and assessed the association of taste receptor variants with indicators of glucose dysregulation, including a diagnosis of type 2 diabetes mellitus and high levels of blood glucose and insulin during an oral glucose tolerance test. We report that a *TAS2R* haplotype is associated with altered glucose and insulin homeostasis. We also found that one SNP within this haplotype disrupts normal responses of a single receptor, TAS2R9, to its cognate ligands ofloxacin, procainamide and pirenzapine. Together, these findings suggest that a functionally compromised TAS2R receptor negatively impacts glucose homeostasis, providing an important link between alimentary chemosensation and metabolic disease.

## Introduction

Taste strongly influences food preference and intake [1]–[3], and taste receptor variants have been associated with differences in taste perception [4]–[6], alcohol consumption [7]–[9] and tobacco use [10]. TAS1R- and TAS2R-type taste receptors are expressed in both the gustatory [11]–[13] and digestive [12], [14] systems, where they play important roles in taste sensation [11], [13] and post-ingestive nutrient responses [14]–[17], respectively. Bitter-tasting compounds activate TAS2R receptors, while taste stimuli that evoke perceptions of sweet or umami (e.g., the taste of glutamate) are detected by receptors of the TAS1R family [11], [12]. Variation in sensitivity to some bitter-tasting molecules has a strong genetic component in humans [12], [18], [19], and in certain cases has been linked to polymorphisms in specific *TAS2R* receptor genes [4], [5], [20]. There is little evidence for interindividual differences in sweet taste sensitivity in humans, though a polymorphism that decreases ligand affinity of mouse Tas1r3 also decreases sweet taste sensitivity [12], [21], [22].

Both TAS1R and TAS2R taste receptors are expressed in the gastrointestinal tract of rodents and humans [14]–[17], [23]–[26]. TAS1Rs mediate nutrient assimilation and other physiological responses to sweet-tasting stimuli [15]–[17], while TAS2Rs may be important for responses to bitter-tasting stimuli [26]. For example, the incretin hormone glucagon-like peptide-1 (GLP-1) is secreted in a taste receptor-dependent manner by gut enteroendocrine L cells in response to stimulation with natural and artificial sweeteners [15]. GLP-1 impacts glucose homeostasis by regulating glucose-stimulated insulin biosynthesis and secretion from pancreatic β-cells and by inhibiting glucagon secretion from pancreatic α-cells [27].

Because of the important role of TAS1R and TAS2R taste receptors in nutrient detection and response in the gustatory and digestive systems, we hypothesized that allelic variations affecting the function of individual TAS1Rs or TAS2Rs could significantly impact glucose homeostasis. We initiated a candidate gene study within the Amish Family Diabetes Study (AFDS) [28], followed by functional characterization of candidate receptor variants, to identify specific sequence variants in *TAS1R* genes and/or *TAS2R* genes that are associated with glucose homeostasis.

## Results

Glucose dysregulation, including elevated plasma glucose, increased hepatic gluconeogenesis, and decreased insulin mediated glucose transport, is a hallmark of type 2 diabetes mellitus (T2DM) [29]. We first asked whether any variants in taste receptor genes are associated with T2DM in the Amish. We genotyped haplotype-tagging, single nucleotide polymorphisms (SNPs) in or around all *TAS1R* and *TAS2R* genes in all T2DM cases (n = 145) and a subset of controls (n = 358) from the Amish Family Diabetes Study (AFDS [28]; **Tables 1** **–** [Table pone-0003974-t002] [Table pone-0003974-t003] **4**). Only four SNPs, all on chromosome 12, showed significant associations with T2DM (**Tables 1** **–** [Table pone-0003974-t002] **3**). Of these, the non-coding SNP rs2588350 showed the greatest significance (P = 0.0007; p_ACT value = 0.025 after correction for multiple comparisons). No SNPs on chromosomes 5 or 7 were associated with T2DM (**Table 2**). Surprisingly, although both the TAS1R2 and TAS1R3 proteins are required for normal glucose sensing in both the gustatory and gastrointestinal systems (e.g., [16], [17], [30]–[32]), we observed no significant associations with *TAS1R* haplotype-related SNPs (**Table 3**). Indeed, all *TAS1R3* SNPs were monomorphic in the Amish (**Table 3**).

**Table 1:** Genotyping statistics for chromosome 12 *TAS2R* SNPs tested in the AFDS.

Chromosome, Position (kb)	SNP ID	Associated/Nearest Gene	Call Rate (%)	HWE *P* Value	Major / Minor Allele	MAF	SNP Type	T2DM Association *P* Value
**12, 10844**	**rs2588350**	***TAS2R7***	**97.3**	**0.679**	**C/T**	**0.07**	**noncoding**	**0.0007**
**12, 10846**	**rs619381**	***TAS2R7***	**94.3**	**0.419**	**C/T**	**0.07**	**M304I**	**0.009**
**12, 10853**	**rs3741845**	***TAS2R9***	**97.4**	**0.013**	**C/T**	**0.12**	**A187V**	**0.005**
12, 10869	rs10845219^B^	*TAS2R10*	70.6	0.254	C/T	0.13	noncoding	N/A
12, 10952	rs1015443^A^	*TAS2R13*	97.5	0.003	C/T	0.21	S259N	N/A
12, 10983	rs7138535	*TAS2R14*	95.4	0.1	T/A	0.08	G38G	0.58
12, 11030	rs10772397^B^	*TAS2R50*	74.6	0.057	T/C	0.22	P259P	N/A
12, 11030	rs1376251	*TAS2R50*	97.4	0.941	C/T	0.25	C203Y	0.99
**12, 11032**	**rs6488334**	***TAS2R50***	**96.5**	**0.197**	**C/T**	**0.12**	**noncoding**	**0.04**
12, 11039	rs10845278^B^	*TAS2R49*	71.8	0.149	T/C	0.50	noncoding	N/A
12, 11042	rs7135018	*TAS2R49*	89.5	0.220	T/C	0.11	K79E	0.08
12, 11042	rs7301234	*TAS2R49*	91.3	0.601	G/A	0.28	noncoding	0.76
12, 11043	rs10772408	*TAS2R49*	94.3	0.576	T/C	0.40	noncoding	0.51
12, 11066	rs10772420	*TAS2R48*	95.6	0.122	A/G	0.34	C299R	0.60
12, 11066	rs1868769^A^	*TAS2R48*	93.4	2.04E-18	A/G	0.17	L140L	N/A
12, 11067	rs4763235	*TAS2R48*	96.3	0.96	C/G	0.25	noncoding	0.95
12, 11073	rs11612527^B^	*TAS2R44*	65.2	0.656	T/A	0.11	noncoding	N/A
12, 11075	rs10845293^A^	*TAS2R44*	95.3	2.50E-88	A/G	0.32	V227A	N/A
12, 11105	rs2708381	*TAS2R46*	92.6	0.243	G/A	0.11	W250#	0.06
12, 11105	rs2708380	*TAS2R46*	97.1	0.107	T/A	0.39	L228M	0.69
12, *n.d.*	rs3759245^A^	*TAS2R45*	93.4	0.001	T/C	0.12	C238R	N/A
12, *n.d.*	rs28581524	*TAS2R45*	91.3	0.160	C/G	0.24	H210Q	0.93
12, 11135	rs35720106^A^	*TAS2R43*	96.5	1.53E-44	C/G	0.24	T221T	N/A
12, 11177	rs2599404	*TAS2R47*	97.1	0.629	C/A	0.36	L252F	0.77
12, 11230	rs1451772^A^	*TAS2R55/42*	95.7	5.27E-06	T/C	0.15	Y265C	N/A
12, 11230	rs5020531	*TAS2R55/42*	96.2	0.025	C/T	0.25	S196F	0.84

Chromosome 12 *TAS2R* SNP found to be monomorphic in the AFDS: rs12578654.

A Excluded from further analysis due to failure of Hardy-Weinberg equilibrium (HWE) expectation (P<0.001).

B Excluded from further analysis due to call rate <90%.

Bold indicates SNPs also reported in Table 2.

kb, kilobases.

*n.d.*, not determined (the Celera genome sequence places *TAS2R45* between *TAS2R46* and *TAS2R42*).

# , stop codon.

MAF, minor allele frequency.

Covariates: age, sex, BMI, and with adjustments for family structure.

**Table 2 pone-0003974-t002:** Genotyping Statistics for chromosome 5 and 7 *TAS2R* SNPs tested in the AFDS.

Chromosome, Position (kb)	SNP ID	Associated/Nearest Gene	Call Rate (%)	HWE *P* Value	Major / Minor Allele	MAF	SNP Type	T2DM Association *P* Value
5, 9681	rs41467	*TAS2R1*	94.9	0.291	G/T	0.47	noncoding	0.98
5, 9682	rs2234233	*TAS2R1*	94.5	0.809	C/T	0.24	R206W	0.91
7, 122420	rs1357949	*TAS2R16*	96.5	0.581	A/G	0.26	noncoding	0.50
7, 122421	rs6466849	*TAS2R16*	97.4	0.966	C/T	0.29	noncoding	0.97
7, 122422	rs860170	*TAS2R16*	94.9	0.089	A/G	0.38	H222R	0.36
7, 122423	rs978739	*TAS2R16*	97.7	0.014	A/G	0.35	noncoding	0.42
**7, 141109**	**rs11763979**	***TAS2R3***	**98.4**	**0.227**	**G/T**	**0.27**	**noncoding**	**0.03**
7, 141111	rs2270009^A^	*TAS2R3*	81.7	0.342	C/T	0.23	G269G	N/A
7, 141111	rs2233998	*TAS2R4*	92.7	0.052	T/C	0.23	F7S	0.08
7, 141125	rs2234001	*TAS2R4*	97.0	0.073	G/C	0.23	V96L	0.08
7, 141137	rs2227264	*TAS2R5*	95.8	0.103	G/T	0.23	S26I	0.10
7, 141319	rs1726866	*TAS2R38*	97.0	0.430	T/C	0.24	V262A	0.07
7, 141320	rs713598^A^	*TAS2R38*	89.2	0.360	G/C	0.21	A49P	N/A
7, 142592	rs4726600	*TAS2R39*	97.7	0.279	G/A	0.25	noncoding	0.33
7, 142630	rs10260248	*TAS2R40*	97.7	0.928	C/A	0.04	S187Y	0.29
7, 142631	rs534126	*TAS2R40*	98.0	0.622	C/T	0.38	noncoding	0.61
7, 142850	rs10241042^A^	*TAS2R60*	64.6	0.068	C/G	0.22	noncoding	N/A
7, 142852	rs4595035	*TAS2R60*	97.7	0.616	C/T	0.35	R310R	0.86
7, 142885	rs1404634^A^	*TAS2R41*	72.9	0.150	G/A	0.43	noncoding	N/A
7, 142885	rs1404635	*TAS2R41*	100	0.577	G/A	0.16	T63T	0.94
7, 142885	rs10278721	*TAS2R41*	97.7	0.653	C/T	0.16	P127L	0.88

Chromosome 7 *TAS2R* SNPs found to be monomorphic in the AFDS: rs13223346 and rs17464086.

A Excluded from further analysis due to call rate <90%.

Bold indicates SNPs also reported in Table 1.

kb, kilobases.

MAF, minor allele frequency.

Covariates: age, sex, BMI, and with adjustments for family structure.

**Table 3 pone-0003974-t003:** Genotyping Statistics for *TAS1R* SNPs tested in the AFDS.

Chromosome, Position (kb)	SNP ID	Linked Gene	Call Rate (%)	HWE *P* Value	Major / Minor Allele	MAF^§^	SNP Type	T2DM Association *P* Value
1, 6546	RS4908563	*TAS1R1*	98.9	0.014	T/C	0.46	intronic	0.65
1, 6562	RS4908932	*TAS1R1*	93.5	0.194	G/T	0.17	noncoding	0.54
1, 19037	RS12036097	*TAS1R2*	97.2	0.112	G/A	0.46	noncoding	0.62
1, 19037	RS12034674^B^	*TAS1R2*	85.9	0.669	C/T	0.29	noncoding	N/A
1, 19040	RS3935570	*TAS1R2*	96.0	0.227	G/T	0.17	intronic	0.77
1, 19042	RS12137730^A^	*TAS1R2*	90.8	0.085	A/C	0.46	intronic	N/A
1, 19043	RS12567264	*TAS1R2*	93.2	0.132	T/A	0.29	Intronic	0.95
1, 19043	RS7534618	*TAS1R2*	97.6	0.148	T/G	0.29	intronic	0.86
1, 19044	RS12408808	*TAS1R2*	97.4	0.526	G/A	0.24	intronic	0.44
1, 19050	RS4076838	*TAS1R2*	93.8	0.525	T/C	0.29	intronic	0.30
1, 19052	RS4920564	*TAS1R2*	95.7	0.191	T/G	0.42	intronic	0.11
1, 19052	RS4920566	*TAS1R2*	96.6	0.378	A/G	0.25	intronic	0.97
1, 19054	RS28470550	*TAS1R2*	93.6	0.109	A/C	0.39	T294T	0.87
1, 19059	RS9701796	*TAS1R2*	96.9	0.866	G/C	0.11	C9S	0.29

*TAS1R* SNPs found to be monomorphic in the AFDS: rs6662276, rs12030791, rs12030797, rs307377, rs10864628, and rs28374389 (All *TAS1R3* SNPs were monomorphic).

A excluded from further analysis due to genotype quality control issues.

B Excluded from further analysis due to call rate <90%.

kb, kilobases.

MAF, minor allele frequency.

Covariates: age, sex, BMI, and with adjustments for family structure.

**Table 4:** Age and BMI values, according to genotype, for AFDS subjects in Table 1.

SNP	Genotype	Age (yrs)	BMI (kg/m^2^)
rs2588350	CC (n = 600)	43.7±0.6	26.8±0.2
	CT/TT (n = 91)	45.6±1.4	27.4±0.5
rs619381	CC (n = 633)	46.0±0.6	27.1±0.2
	CT/TT (n = 85)	47.6±1.6	27.3±0.5
rs3741845	CC (n = 538)	43.4±0.6	26.8±0.2
	CT/TT (n = 155)	46.3±1.1	27.1±0.4

BMI, body-mass index.

Values are mean±std error.

There is a significant difference in age across genotype (P = 0.02) between CC and CT/TT individuals for rs3741845.

**Figure 1:**
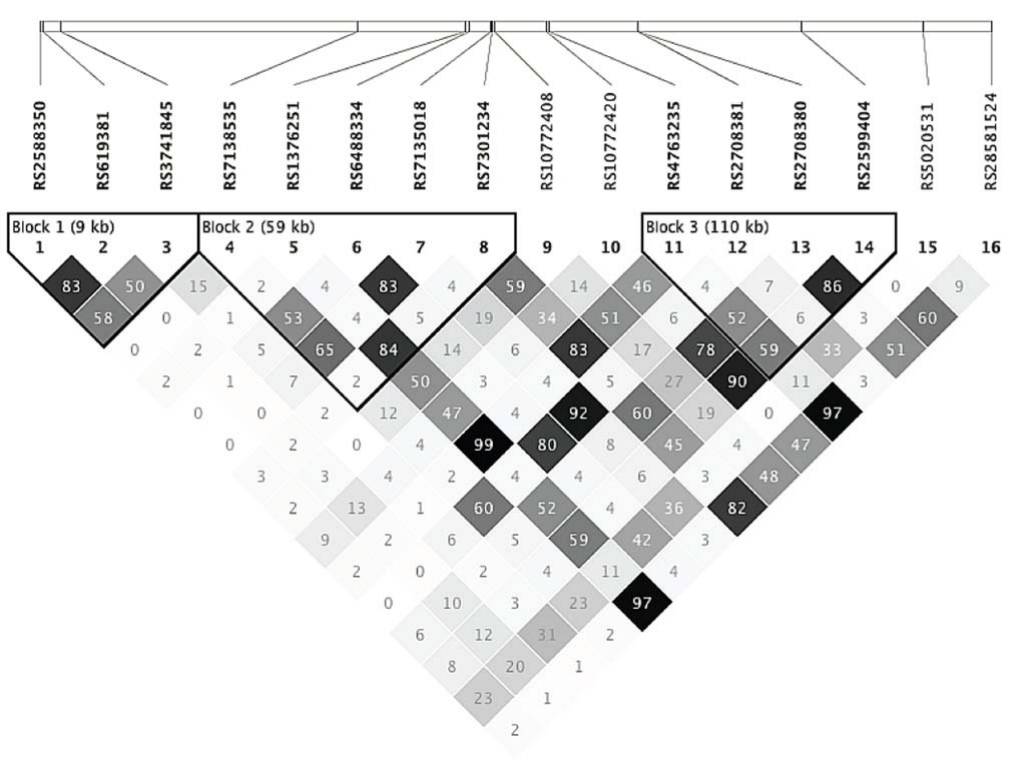
Haplotype structure of TAS2R SNPs on chromosome 12 in the AFDS. Pairwise LD (r^2^) among 16 SNPs within the *TAS2R* cluster on chromosome 12. The relative position of rs3759245 is based on a non-reference assembly (Celera). r^2^ values ×100 are indicated within squares, and with darker shades indicating higher r^2^ values.

Next, we defined the extent of linkage disequilibrium (LD) within the chromosome 12 *TAS2R* cluster. The cluster extends for 380 kb and contains three LD blocks (**Figure 1**). LD Block 1 contains rs2588350 along with two other SNPs with significant T2DM associations (**Table 1**; r^2^ = 0.50–0.83): rs619381, a nonsynonymous coding SNP in *TAS2R7* (C519T, encoding Met304Ile; P = 0.009; p_ACT = 0.24) and rs3741845, another nonsynonymous coding SNP in *TAS2R9* (C560T, encoding Ala187Val; P = 0.005; p_ACT = 0.15). Two haplotype-tagging SNPs, rs797172 and rs812761, in the gene proximal to the *TAS2R* cluster (*CSDA*) show neither significant association with T2DM (P = 0.43 and P = 0.24, respectively) nor LD (r^2^≤0.16) with the *TAS2R* SNPs. These data suggest that a single LD block, containing three TAS2R-tagging SNPs, is the principal taste receptor-related locus for T2DM risk in the Amish.

To confirm that the T2DM association of these three SNPs reflects an underlying dysregulation of glucose and insulin homeostasis, we performed association analyses with glucose and insulin traits obtained in 693 non-diabetic AFDS subjects who had been given a standard 3-hour OGTT. The minor alleles of all three SNPs were significantly associated with several measures of glucose and insulin homeostasis (**Table 5**), including glucose area-under-the-curve (AUC) and insulin response. Insulin AUC was higher for individuals with the minor allele of any of the three SNPs, but these differences were only statistically significant for rs3741845 and rs2588350 (**Table 5**). Estimates of insulin resistance based on homeostatic model assessment were also significantly affected in subjects with the rs3741845 T allele. Thus, the minor alleles of rs2588350, rs619381 and rs3741845 display similar phenotypic associations. As these three SNPs display significant LD (**Figure 1**), we conclude that the minor alleles of these SNPs comprise a single haplotype associated with dysregulated postprandial glucose homeostasis.

**Table 5:** Associations with insulin and glucose metrics from OGTT in non-diabetic AFDS subjects.

Trait	rs2588350 (CC)	rs2588350 (CT/TT)	*P* value	rs619381 (CC)	rs619381 (CT/TT)	*P* value	rs3741845 (CC)	rs3741845 (CT/TT)	*P* value
Glucose 30 min (mmol/l)	8.45±0.18 (n = 636)	8.95±0.19 (n = 97)	**0.006**	8.49±0.19 (n = 614)	9.03±0.20 (n = 84)	**0.005**	8.46±0.12 (n = 568)	8.96±0.15 (n = 167)	**0.0014**
Glucose 60 min (mmol/l)	8.24±0.23 (n = 632)	8.76±0.25 (n = 97)	**0.029**	8.29±0.25 (n = 610)	8.89±0.27 (n = 83)	**0.016**	8.26±0.16 (n = 564)	8.72±0.19 (n = 166)	**0.0006**
Glucose 90 min (mmol/l)	6.54±0.22 (n = 634)	6.99±0.24 (n = 96)	**0.045**	6.55±0.23 (n = 612)	7.15±0.25 (n = 84)	**0.01**	6.58±0.15 (n = 568)	6.90±0.18 (n = 166)	**0.012**
Glucose 120 min (mmol/l)	5.31±0.18 (n = 646)	5.71±0.20 (n = 97)	**0.03**	5.31±0.19 (n = 625)	5.73±0.21 (n = 83)	**0.03**	5.35±0.12 (n = 578)	5.48±0.15 (n = 167)	0.054
GAUC (mmol/l)	19.9±0.2 (n = 600)	21.3±0.4 (n = 90)	**0.043**	18.80±0.44 (n = 580)	19.89±0.47 (n = 84)	**0.01**	19.8±0.2 (n = 538)	21.0±0.3 (n = 155)	**0.036**
Insulin Response (pmol/l)	424.78±46.63 (n = 593)	548.74±50.55 (n = 91)	**0.007**	455.31±46.42 (n = 573)	533.14±49.48 (n = 79)	0.09	426.52±32.09 (n = 532)	512.67±38.94 (n = 155)	**0.0086**
Ln Insulin 30 min (pmol/l)	5.61±0.07 (n = 630)	5.71±0.07 (n = 95)	0.12	5.63±0.07 (n = 608)	5.65±0.07 (n = 83)	0.76	5.60±0.05 (n = 562)	5.71±0.05 (n = 165)	**0.017**
Ln Insulin 60 min (pmol/l)	5.71±0.07 (n = 625)	5.77±0.07 (n = 96)	0.54	5.75±0.07 (n = 603)	5.76±0.07 (n = 84)	0.86	5.71±0.05 (n = 557)	5.79±0.06 (n = 166)	0.1
Ln Insulin 90 min (pmol/l)	5.33±0.07 (n = 627)	5.51±0.07 (n = 96)	**0.012**	5.37±0.07 (n = 605)	5.49±0.07 (n = 84)	0.07	5.33±0.05 (n = 561)	5.47±0.06 (n = 165)	**0.0088**
Ln Insulin 120 min (pmol/l)	4.84±0.08 (n = 635)	5.02±0.08 (n = 95)	**0.024**	4.87±0.08 (n = 615)	4.98±0.07 (n = 83)	0.13	4.85±0.05 (n = 568)	4.95±0.06 (n = 165)	**0.046**
IAUC (mmol/l)	739.8±18.0 (n = 593)	889.8±64.9 (n = 91)	**0.007**	649.30±50.21 (n = 573)	731.07±53.57 (n = 79)	0.10	739.2±19.4 (n = 532)	858.2±44.2 (n = 155)	**0.006**
Ln HOMA	0.85±0.05 (n = 680)	0.92±0.05 (n = 102)	0.23	0.87±0.04 (n = 656)	0.91±0.05 (n = 90)	0.33	0.85±0.03 (n = 604)	0.92±0.04 (n = 176)	**0.035**

Values expressed as mean±standard error, with n = number of subjects.

Covariates: age, sex and BMI, and with adjustments for family structure.

GAUC: glucose area under the curve.

IAUC: insulin area under the curve.

Insulin Response = (Insulin AUC)−3(Insulin at time 0).

Ln HOMA = natural log [(Insulin−10 min)(fasting glucose)/22.5].

**Figure 2:**
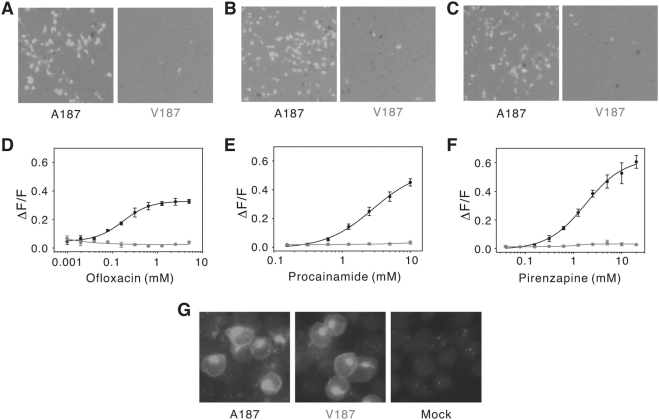
Differential activity of TAS2R9 alleles. (A–C) Calcium imaging assay of TAS2R9 Ala187 and Val187 variants responding to ofloxacin (5 mM) (A), pirenzepine (20 mM) (B) and procainamide (10 mM) (C). (D–F) Dose-response functions of TAS2R9 Ala187 (black) and Val187 (red) variants to ofloxacin (D), pirenzepine (E) and procainamide (F). Error bars are s.e.m. (G) Immunofluorescence staining of HEK293 cells transfected with TAS2R9 Ala187, TAS2R9 Val187 or empty vector (mock).

Though any of the three SNPs within this glucose dysregulation haplotype could potentially affect receptor expression or function, and thus glucose and insulin homeostasis, the rs3741845 T allele is a particularly attractive candidate risk allele: this SNP alters an amino acid within a region of TAS2R9 that is predicted to influence ligand binding and response of other GPCRs, including TAS2Rs [33]. Therefore, we asked whether the Ala to Val change alters the ligand response of TAS2R9. Since TAS2R9 was an orphan receptor, we utilized a high-throughput screening strategy to identify bitter-tasting stimuli that activate TAS2R9. Three of the 64 bitter-tasting compounds screened (Supplemental data, **Table S1**) activated TAS2R9 Ala187-expressing cells: the fluoroquinolone antibiotic ofloxacin (**Figure 2A and D**), the tricyclic gastric acid inhibitor pirenzapine (**Figure 2B and E**) and the antiarrhythmic drug procainamide (**Figure 2C and F**). They did so with an EC50 of 0.2, 1.8 and 2.8 mM, respectively. The Val187 variant of TAS2R9 showed a dramatic loss of function, with no responses to any of the bitter stimuli, even at high concentrations (**Figure 2D, E and F** and data not shown). This functional decrement is not due to differences in surface expression (**Figure 2G**). Thus, the rs3741845 minor allele (T) causes a major functional deficit in ligand response of TAS2R9.

**Figure 3:**
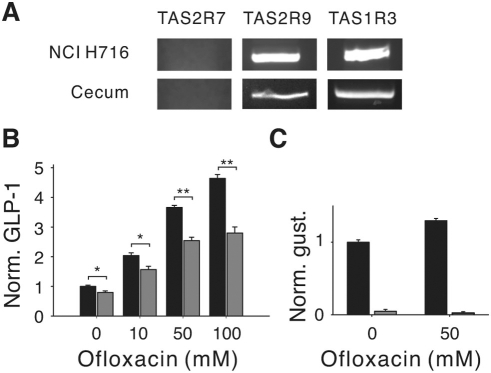
TAS2R9 in enteroendocrine cells. (A) PCR amplicons for TAS2R9 or TAS1R3 from NCI-H716 and human cecum cDNA. The size of the TAS1R3 amplicon (434 bp) indicates no genomic DNA contamination (the genomic product would be 693 bp). TAS2R7 was not amplified from either cDNA pool. (B) GLP-1 secretion from NCI H716 cells in response to ofloxacin stimulation, normalized to the buffer only control, in the absence (black) or presence (red) of an α-gustducin siRNA. The specificity of the siRNA probe for α-gustducin in these cells was previously reported [15]. Repeated measures ANOVA showed significant effects of concentration (*P*<1×10^−9^), siRNA treatment (*P* = 1.4×10^−5^) and siRNA treatment X concentration (*P* = 9×10^−5^). Posthoc *t*-tests: * *P*<0.05; ** *P*<0.001. (C) Levels of α-gustducin message in NCI H716 cells measured by quantitative real-time PCR in the absence (black) or presence (red) of the α-gustducin siRNA and normalized for α-gustducin levels in the absence of stimulus and siRNA. Error bars: standard error.

Though the mechanism by which this taste receptor-associated haplotype affects glucose and insulin homeostasis remains unclear, these receptors could be involved in the modulation of GLP-1 secretion from gut enteroendocrine L cells [14]. We used reverse transcription-polymerase chain reaction (RT-PCR) to determine if TAS2R9 is expressed in these cells. We amplified both TAS2R9 and TAS1R3 (a subunit of the sweet and umami taste receptors previously reported to be expressed in enteroendocrine L cells [15], [23], [25]) from cDNA obtained from NCI-H716 cells (a human enteroendocrine L cell line; **Figure 3A**), from human cecum (**Figure 3A**), and from human tongue (data not shown). We were unable to amplify TAS2R7 from any of these cDNA pools (**Figure 3A** and data not shown), though we could amplify a product from human genomic DNA (data not shown). The TAS2R9 products were amplified from cDNA and not genomic DNA contaminants: PCR from control samples that were not reverse transcribed gave no TAS2R9 product (data not shown), and oligos that recognize coding sequences in exons 4 and 6 of taste receptor TAS1R3 amplify a product lacking the two intervening introns (**Figure 3A**). Independent clones of the TAS2R9 product amplified from the NCI-H716 cells had either an A or T at bp 560, indicating that this cell line is heterozygous for this allele. Next, we tested whether a TAS2R9 ligand can promote GLP-1 secretion from enteroendocrine L cells. Stimulation of NCI-H716 cells with ofloxacin elicited a concentration-dependent secretion of GLP-1 from this cell line (**Figure 3B**). siRNA knockdown of the G protein α-gustducin (**Figure 3C**), which mediates bitter taste responses in the tongue [34] and which has been implicated in taste receptor-mediated GLP-1 secretion in the gut [15], reduced ofloxacin-stimulated GLP-1 secretion (**Figure 3B**). Together, these results are consistent with a role of TAS2R9 in the regulation of nutrient-dependent GLP-1 secretion from L cells.

## Discussion

By combining human genetic approaches with high-throughput receptor screening, we have identified an important link between taste receptor function and the modulation of glucose homeostasis. Our study provides genetic and biological validation of an association between a TAS2R haplotype on human chromosome 12 with the regulation of glucose and insulin levels. The novel role of TAS2Rs in the maintenance of glucose homeostasis should help elucidate the relative contributions of taste receptor-mediated chemoreception in the gustatory and digestive systems and suggests new lines of investigation for ameliorating risk of metabolic disease and for developing novel avenues for treatment.

Our conclusions are foremost based on the genetic association of a TAS2R haplotype, including the TAS2R9 T560 allele, with measures of glucose and insulin dysregulation in non-diabetic Amish individuals (**Table 5**) and with increased incidence of T2DM in a case-control group (**Tables 1** **–** [Table pone-0003974-t002] [Table pone-0003974-t003] **4**). The observed concordance of several related but independent phenotypic measures (e.g., measures of glucose response during an OGTT, measures of insulin response during an OGTT, and T2DM diagnosis) with the same allele provides important internal replication of the association. The few T2DM genome-wide association studies (GWASs) that included rs619381 and rs3741845 (but not rs2588350) [35]–[37] do not report significant associations between T2DM and these SNPs. However, the conservative cutoffs necessary for GWAS (∼5×10^−7^) would likely exclude these associations from consideration in most studies. Indeed, one heavily replicated T2DM gene, *PPARG*, would not have met the criteria for a novel T2DM gene in at least one GWAS [38]. The AFDS, though a relatively small study in a homogeneous population, has exhibited sufficient power to identify or replicate at least two T2DM risk alleles that are replicated in other populations [39], [40]. However, association studies of rs3741845 in genetically heterogeneous populations may be further complicated by population stratification, as the frequency of the rs3741845 alleles varies greatly across human populations (Supplementary data, **Table S2**). Further genetic and mechanistic analyses will be needed to determine the extent to which contributions of specific TAS2R variants to glucose dysregulation are found in other populations.

We also provide important biological validation of the association data: a physiological consequence of the TAS2R9 polymorphism (i.e., a loss of response to several ligands). The rs3741845 SNP predicts an amino acid change in the second extracellular loop or fifth transmembrane domain of TAS2R9, a region suggested to form part of the ligand binding pocket and to be important for receptor activation [33], [41], [42]. In contrast, rs619381 affects an amino acid in the C-terminal domain of TAS2R7, a region unlikely to directly impact ligand interactions, and the rs2588350 SNP is a non-coding polymorphism. While any of these SNPs could potentially impact TAS2R expression or function, and therefore glucose and insulin homeostasis, we reasoned that the TAS2R9 variant was the most likely to significantly alter receptor function. The observation that TAS2R9, but not TAS2R7, is expressed in human enteroendocrine cells (**Figure 3**) further supports a key role for TAS2R9. The single amino acid change from Ala to Val at position 189 has a profound effect on TAS2R9 function, abolishing responses to three different ligands (**Figure 2**). No systematic structure-function analyses have been performed for TAS2Rs, and studies that can differentiate ligand binding from other aspects of receptor activation have been limited to TAS1Rs (e.g., [22], [43], [44]). Though we cannot resolve whether the Ala to Val change specifically impacts ligand binding, binding-induced conformational changes, or effective G protein coupling, the inability of the Val187 variant to respond to any of three different ligands provides compelling evidence that this variant is incapable of transducing stimuli.

The observation that TAS2R9 is expressed in enteroendocrine cells and that a TAS2R9 ligand can elicit GLP-1 secretion suggests a possible mechanism, regulation of incretin response in the gut, by which variation in taste receptor function could impact glucose and insulin regulation. Gut TAS2Rs could be stimulated by a number of compounds, including ingested toxins or bitter-tasting peptides that result from the fermentation of proteins such as casein [45]. Gut flora, which can vary dramatically between obese and lean individuals [46], [47], could also serve as a source of TAS2R stimuli in normal or pathogenic states. Many TAS2Rs are broadly tuned to multiple stimuli, and some bitter stimuli activate more than one TAS2R [12], [20], [41], [42], [48]–[50]. The three TAS2R9 ligands identified in this study, ofloxacin, pirenzapine and procainamide, are not natural ligands for this receptor, some of which would be expected to activate TAS2R9 with a higher efficacy. Even so, they do serve as effective tools to assay the consequences of the Ala187Val mutation (**Figure 2**). Interestingly, some fluoroquinolones, particularly gatifloxacin and levofloxacin (the L-isomer of ofloxacin), have been associated with dysglycemia in diabetic and non-diabetic patients [51]. TAS2R9 does not respond to the three other fluoroquinolones we tested (gatifloxacin, ciprofloxacin and enoxacin; Supplementary data, **Table S1**), but it is intriguing to consider whether some bitter-tasting pharmaceuticals may affect glucose homeostasis, at least in part, through actions on TAS2Rs.

However, we cannot rule out alternative physiological mechanisms that link TAS2R function to the modulation of glucose homeostasis. For example, taste receptors could affect glucose homeostasis through a gustatory mechanism by altering the perceived qualities of food and impacting food preference and intake [6], [30]. Indeed, taste receptor polymorphisms affect the ability to recognize taste stimuli by altering the perceived qualities of food and impacting food preference and intake [1], [6]. Intragastric infusion of sweet- and bitter-tasting compounds also impacts taste preference [52], [53]. Therefore, blindness to particular bitter-tasting compounds could lead to increased ingestion of toxins [3]; alternatively, hypersensitivity could result in avoidance of otherwise beneficial foods (for example, individuals with the phenylthiocarbamide-sensitive version of TAS2R38 are more sensitive to the bitterness of certain vegetables [6]). It is also unclear to what extent the unique lifestyle and history of the Amish impacts the contribution of TAS2R variants to manifestations of dysregulated glucose and insulin homeostasis, including the development of insulin resistance and T2DM. In any case, our studies reveal that bitter taste receptors can influence glucose and insulin homeostasis. The novel role of TAS2Rs in maintenance of glucose homeostasis should help elucidate the relative contributions of taste receptor-mediated chemoreception in diverse alimentary tissues and suggests new lines of investigation for ameliorating risk of metabolic disease and for developing novel avenues for treatment.

## Materials and Methods

### Subjects

The University of Maryland School of Medicine's Institutional Review Board approved all studies. The Amish Family Diabetes Study (AFDS) is an ongoing effort to identify genetic contributors to obesity, diabetes, cardiovascular disease and related disorders [28], [39], [54]. Detailed descriptions of the population (the Old Order Amish of Lancaster County, Pennsylvania, USA), study design, recruitment methods, phenotypic characterization, clinical characteristics of the subjects and statistical methods have been published previously [28]. Informed consent, including permission to contact relatives, was obtained before participation [28]. In brief, probands were defined as individuals with previously diagnosed diabetes (age of diagnosis between 35 and 65 years). First- and second-degree relatives of the probands were also recruited, as were first- and second-degree relatives of any additional diabetic individuals identified. Currently, the AFDS includes over 1300 subjects. Participants in the AFDS, the Old Order Amish of Lancaster, Pennsylvania, have a common lifestyle and socioeconomic status, and possess detailed genealogical records dating to the period of their early migration from Europe in the 1700's [28].

### Genotyping

We identified candidate haplotype tagging SNPs (r^2^≥0.8) from the HapMap [55] and additional SNPs in coding and regulatory regions from the Entrez SNP database [56] and from the literature [4], [8], [57], [58]. In total, 70 *TAS1R*- and *TAS2R*-associated SNPs were genotyped in the AFDS. Forty-five of these SNPs were polymorphic in the AFDS and passed quality control filters and were subsequently analyzed (see below and **Tables 1** **–** [Table pone-0003974-t002] **3**). All SNPs were genotyped using the TaqMan platform (Applied Biosystems) according to manufacturer's protocols. SNPs found to be monomorphic in the AFDS (*n* = 9) were not analyzed further. Genotypes were checked for Mendelian consistency; inconsistencies, which were detected in <0.5% of genotypes, were removed from analysis. Genotype frequencies of all SNPs were tested for consistency with Hardy–Weinberg expectations by the χ^2^ test. Markers that showed extreme deviation from Hardy-Weinberg Equilibrium in controls (*P*<0.001) were eliminated from further analysis (*n* = 7), as were SNPs with call rates <90% (*n* = 9).

### Heterologous expression and functional assay

Receptor expression and functional assays were performed as previously described [20], [49]. We used FLIPR (Molecular Devices) to screen the function of TAS2R9 and to establish dose-response curves for the tested compounds (Supplementary data, **Table S1**). We cloned the cDNAs encoding the *TAS2R9* Ala^187^ and Val^187^ variants into a pEAK10-derived vector (Edge Biosystems, Gaithersburg, MD). The vector was engineered to generate translational fusion to the N-terminus of the rat somatostatin type 3 receptor (45 amino acids), and the C-terminus of the herpes simplex virus (HSV) glycoprotein D epitope, as described [49]. Immunocytochemistry was performed using antiserum against HSV glycoprotein D (Novagen, 1∶10,000), as described [49], except the secondary antibody was a FITC-conjugated donkey antiserum against mouse IgG (Molecular Probes, 1∶1,000).

Plasmids containing *TAS2R9* cDNAs were transiently transfected into HEK293 cells stably expressing the chimeric G protein subunit G〈_16gust44_ [59] using TransIT-293 (Mirus Corporation), according to the manufacturer's protocol. Cells were plated into 384- well plates and after 24–30 hr loaded for 1 h with the calcium-sensitive dye Fluo4-AM and stimulated with bitter compounds. Calcium signals were recorded simultaneously from each well after excitation at 488 nm. The obtained signals (F) were normalized to the fluorescence of cells before stimulation (Fo) and expressed as ΔF/F value: ΔF/F = (F−Fo) / Fo. Responses of four wells containing cells expressing the same receptor and receiving the same stimulus were averaged. Calculations were based on at least three independent transfection experiments.

### Reverse transcription PCR

Total RNA was isolated from human enteroendocrine NCI-H716 cells with Trizol reagent, then reverse transcribed with random hexamer probes. A reaction without reverse transcriptase was included to control for genomic DNA contamination. Human cecum cDNA was obtained from Biochain Institute (Hayward, CA). *TAS2R7* (GeneID: 50837) and *TAS2R9* (GeneID: 50835) gene specific primers recognized the single coding exons of each gene. *TAS1R3* (GeneID: 83756) gene specific primers were directed against exons 4 and 6. All PCR products were verified by sequencing.

### GLP-1 assays

Human enteroendocrine NCI-H716 cells were maintained and assayed for GLP-1 secretion as described by Jang et al. [15] in the presence or absence of ofloxacin (Sigma Chemical) at 10, 50 or 100 mM in phosphate buffered saline (PBS), pH 7.2. Control samples received PBS only. GLP-1 was measured by ELISA and normalized to protein content. For siRNA knockdown experiments, an α-gustducin-specific siRNA (see [15]) was transfected into subconfluent NCI-H716 cells 48 hr prior to ofloxacin stimulation and GLP-1 secretion analysis. Reduction of α-gustducin message was verified by quantitative real time PCR. The efficacy of the stimulation was significantly reduced after knockdown of the G protein α-gustducin by RNA interference (**Figure S1B, C**), indicating that ofloxacin-dependent GLP-1 secretion is mediated by a G protein-coupled receptor.

### Statistical Analysis

Associations with SNP genotype and the various phenotypes were performed using pedigree-based analysis by regressing the effect of the marker genotype while accounting for residual familial correlations among related individuals using age, sex, and body mass index (BMI) as covariates (age and BMI are positively correlated with T2DM in the AFDS). To account for the relatedness among family members, we employed the measured genotype approach, in which we estimated the likelihood of specific genetic models given the pedigree structure. Parameter estimates were obtained by maximum likelihood methods and the significance of association was tested by likelihood ratio tests. When discrete outcome traits were analyzed, a threshold model was assumed. All analyses of the AFDS were carried out using the Sequential Oligogenic Linkage Analysis Routines (SOLAR) software program [60]. In the T2DM case/control analysis, a recessive genetic model was assumed. When analyzing data from non-diabetic AFDS subjects, a dominant model was assumed. To control for an inflation in the type I error rate due to the number of comparisons in our initial T2DM association analyses, we use the P_ACT_ statistic [61], which attains the accuracy of permutation or simulation-based correction through the adjustment of correlated p-values. Unadjusted P values are reported in all tables.

Pairwise LD between the SNPs and haplotype block analysis was computed using Haploview 4.0 [62]. Haplotype blocks were defined by 95% confidence bounds on D' [63]. Concentration-response curves and EC50 values derived from the heterologous expression and functional assays were calculated in SigmaPlot by nonlinear regression.

## Supporting Information

Table S1 (0.05 MB PDF)

Table S2 (0.06 MB PDF)
