# Supplementary material for: Bitter Taste Receptors Influence Glucose Homeostasis
Source: PLoS One. 2008 Dec 18;3(12):e3974. doi: 10.1371/journal.pone.0003974 (PMC2597743; doi:10.1371/journal.pone.0003974)
Supplement: Table S1 — (0.05 MB PDF) [file pone.0003974.s001.pdf]

Table S1 | Bitter tasting compounds that did not activate TAS2R9

Compound	Highest Conc. Tested (mM)
Acetylpyrazine	10
Acetaminophen	10
Aloin	0.1
Amygdalin	10
Andrographolide	0.5
Arbutin	5
Aristocholic Acid	0.02
Atropine	1
Brucine	1
Cinchonine	0.6
Ciprofloxacin	0.5
Clarithromycin	0.5
Clindamycin	1.6
Cycloheximide	10
Cyclooctanone	20
Dexamethasone	1
Diltiazem	0.8
Diisobutylamine	4
Dimethylbiguanide	100
2,6- Dimethylpiperidine	2.5
Doxepin	0.3
Enalapril	20
Edrophonium	40
Enoxacin	1
Epicatechin	6
Erythromycin	0.8
Ethylpyrazine	20
Ethyl Benzoate	12.5
Ethylene Thiourea	4.5
Gatifloxacin	4
Ginkgolide A	2
Goitrin	0.8
Guaiacol glyceryl ether	5
Labetalol-HCl	0.5
Lomefloxacin	3
Lupinine	5
N-Methylthiourea	40
1-methy-2-quinolinone	5
6〈-methylprdnisolone	0.4
Nitronaphthalene	0.15
Oleuropein	5
Omeprazole	0.5
N'-Ethyl-N'5-Phenylurea	10
Phenylthiocarbamide (PTC)	2
Picoline	10
Prednisone	0.5
Propylthiouracil (PROP)	2
Quassin	0.2
Quinacrine	0.04
Salicin	20
Sodium Saccharin	0.01
Spartein Sulfate Pentahydrate	0.8
Sucrose Octaacetate	0.2
Sulfamethoxazole	2.5
Thioacetanilide	0.15
Thiocarbanilide	0.5
Tolazoline	20
Tolylurea	2.5
Trapidil	1.5
Trimethoprim	1.5
Zyrtec	1
