# Supplementary material for: Bitter Taste Receptors Influence Glucose Homeostasis
Source: PLoS One. 2008 Dec 18;3(12):e3974. doi: 10.1371/journal.pone.0003974 (PMC2597743; doi:10.1371/journal.pone.0003974)
Supplement: Table S2 — (0.06 MB PDF) [file pone.0003974.s002.pdf]

Table S2 | Frequency of the T allele of rs3741845 in different populations

Population	Frequency	Source
AFDS	0.11	This paper
JPT ‡	0.24	International HapMap Consortium [1]
Japanese	0.25	Kim *et al.*, 2005 [2]
CEU †	0.35	International HapMap Consortium [1]
Hungarian	0.56	Kim *et al.*, 2005 [2]
African American	0.65	Entrez SNP [3]
Pygmy	0.70	Kim *et al.*, 2005 [2]
YRI §	0.77	International HapMap Consortium [1]
Amerindian	0.80	Kim *et al.*, 2005 [2]
Cameroonian	0.86	Kim *et al.*, 2005 [2]

†, Utah residents of European Ancestry
‡, Japanese in Tokyo, Japan
§, Yoruba in Ibadan, Nigeria

1. The International HapMap Consortium (2005) A haplotype map of the human genome. Nature 437: 1299-1320.
2. Kim U, Wooding S, Ricci D, Jorde LB, Drayna D (2005) Worldwide haplotype diversity and coding sequence variation at human bitter taste receptor loci. Hum Mutat 26: 199-204.
3. Sherry ST, Ward MH, Kholodov M, Baker J, Phan L, et al. (2001) dbSNP: the NCBI database of genetic variation. Nucleic Acids Res 29: 308-311.
